# Evaluation of eight live attenuated vaccine candidates for protection against challenge with virulent *Mycobacterium avium* subspecies *paratuberculosis* in mice

**DOI:** 10.3389/fcimb.2014.00088

**Published:** 2014-07-01

**Authors:** John P. Bannantine, Jamie L. Everman, Sasha J. Rose, Lmar Babrak, Robab Katani, Raúl G. Barletta, Adel M. Talaat, Yrjö T. Gröhn, Yung-Fu Chang, Vivek Kapur, Luiz E. Bermudez

**Affiliations:** ^1^Infectious Bacterial Diseases, National Animal Disease Center, USDA-ARSAmes, IA, USA; ^2^Department of Biomedical Sciences, College of Veterinary Medicine, Oregon State UniversityCorvallis, OR, USA; ^3^Department of Microbiology, College of Science, Oregon State UniversityCorvallis, OR, USA; ^4^Department of Veterinary and Biomedical Sciences, Pennsylvania State UniversityUniversity Park, PA, USA; ^5^School of Veterinary Medicine and Biomedical Sciences, University of NebraskaLincoln, NE, USA; ^6^Department of Pathobiological Sciences, University of Wisconsin-MadisonMadison, WI, USA; ^7^Department of Food Hygiene, Cairo UniversityCairo, Egypt; ^8^Department of Population Medicine and Diagnostic Sciences, College of Veterinary Medicine, Cornell UniversityIthaca, NY, USA

**Keywords:** Johne's disease, *Mycobacterium*, vaccines, attenuated, mouse model, genomics

## Abstract

Johne's disease is caused by *Mycobacterium avium* subsp. *paratuberculosis* (MAP), which results in serious economic losses worldwide in farmed livestock such as cattle, sheep, and goats. To control this disease, an effective vaccine with minimal adverse effects is needed. In order to identify a live vaccine for Johne's disease, we evaluated eight attenuated mutant strains of MAP using a C57BL/6 mouse model. The persistence of the vaccine candidates was measured at 6, 12, and 18 weeks post vaccination. Only strains 320, 321, and 329 colonized both the liver and spleens up until the 12-week time point. The remaining five mutants showed no survival in those tissues, indicating their complete attenuation in the mouse model. The candidate vaccine strains demonstrated different levels of protection based on colonization of the challenge strain in liver and spleen tissues at 12 and 18 weeks post vaccination. Based on total MAP burden in both tissues at both time points, strain 315 (MAP1566::Tn*5370*) was the most protective whereas strain 318 (intergenic Tn*5367* insertion between MAP0282c and MAP0283c) had the most colonization. Mice vaccinated with an undiluted commercial vaccine preparation displayed the highest bacterial burden as well as enlarged spleens indicative of a strong infection. Selected vaccine strains that showed promise in the mouse model were moved forward into a goat challenge model. The results suggest that the mouse trial, as conducted, may have a relatively poor predictive value for protection in a ruminant host such as goats.

## Introduction

The development of vaccines for Johne's disease, which affects cattle, sheep, and goats, is an attractive approach to control this disease. The ideal vaccine would stimulate a protective immune response to *Mycobacterium avium* subspecies *paratuberculosis* (MAP), the cause of this disease, and will not interfere with diagnostic tests. This would have a significant impact, given the wide distribution of the disease in Europe, Australia, New Zealand, Japan, India, and the United States. Furthermore, this condition is economically crippling in farmed livestock operations. Outdated figures from the National Animal Health Monitoring System suggested the prevalence of Johne's disease in U.S. dairy herds to be 68% and the cost to the industry to be approximately $250 million annually (Johnson-Ifearulundu et al., 1999). More recent data suggest this prevalence is on the rise with approximately 91% of dairy herds infected (Lombard et al., 2013).

Vaccination against MAP has been found to reduce the incidence of clinical disease, although animals are still susceptible to infection with MAP (Larsen et al., 1978). While there are significantly fewer bacteria in the intestinal tissues of vaccinated calves vs. non-vaccinated (Sweeney et al., 2009), perhaps the most tangible benefit to vaccination is that it lowers bacterial shedding levels in the feces. This helps decrease transmission to uninfected herd mates, which has been demonstrated more than once (Kormendy, 1994; Sweeney et al., 2009), but most recently by Knust et al. using a killed whole cell vaccine (Knust et al., 2013).

This study represents the second phase of a three-phase vaccine trial to identify the best available live attenuated vaccine candidates against Johne's disease. These strains were tested for attenuation in primary bovine macrophages in the first phase and prior to enrollment in the current study. All MAP mutants were attenuated to some degree in a primary bovine macrophage model (Lamont et al., 2014); however, only the eight most attenuated in macrophages were evaluated in the current mouse study. The goal of this study was to determine the protective efficacy of eight MAP deletion mutants in C57BL/6 mice. Bacterial load in the spleen and liver were determined following vaccination and subsequent challenge with MAP. Five vaccine candidates showing the least amount of viable bacteria in these tissues were moved forward into phase three of the study, the goat trial (Hines et al., 2014).

Historically, live MAP vaccine formulations were attenuated by serial passage on solid media (Huygen et al., 2010). The vaccine currently used in the United States is based on a killed whole-cell bacterin derived from *M. avium* strain 18 in an oil adjuvant and sold under the name Mycopar. More recently, the introduction of random transposon insertions was achieved using mycobacteriophage and the first library of 5620 Tn*5367* insertional mutants was reported (Harris et al., 1999). Screening transposon library banks for virulence genes (Shin et al., 2006) and attenuation in animals (Scandurra et al., 2010) quickly followed. But it was only recently that a few laboratories have been able to construct defined knockouts by allelic exchange with this bacterium, which is slow growing and not easily amenable to genetic manipulation (Park et al., 2008; Scandurra et al., 2010; Chen et al., 2012). Although the macrophage trial mentioned above contains mutants constructed by allelic exchange, this communication only reports on transposon insertion mutants.

The ability to construct mutant strains has now made feasible the testing of live attenuated bacterial clones vs. heat killed preparations of MAP to determine which yields the most protective vaccine. This comparison was recently performed using the Mycopar vaccine and a live attenuated *leuD* mutant in mice. In that study, the live vaccine was more protective than the killed vaccine (Faisal et al., 2013). All commercially available MAP vaccine formulations include heat-inactivated mycobacteria and are sold under the Mycopar, Silirum, and Gudair trade names. Silirum and Mycopar are available, but not used in the United States while Gudair is used in Europe and Australia. In the current study, the heat-inactivated prep, Silirum, was used as a comparison to the live attenuated mutants. Silirum has recently been shown to lower clinical disease in New Zealand commercial deer farms; however no differences in weight gain or fecal shedding were observed between vaccinates and controls (Stringer et al., 2013).

## Materials and methods

### Bacterial cultures

MAP bovine strain K-10 was used as the challenge strain. It was cultured in Middlebrook 7H9 broth supplemented with 10% (v/v) oleic acid, albumin, dextrose, and catalase (OADC; Hardy, Santa Maria, CA) and 2 mg/L mycobactin J (Allied Monitor, Fayette, MO) at 37°C. Cultures were centrifuged and washed 3 times in phosphate-buffered saline (PBS; 150 mM NaCl, 10 mM NaPO4, pH 7.4) before bacterial pellets were resuspended to make a final 10^8^ MAP/ml suspension to be used as the challenge inoculum. Strain 315 was derived by random transposon mutagenesis of wild type strain K-10 with Tn*5370*, whereas strains 316–321 were derived by random transposon mutagenesis of K-10 with Tn*5367* (Table 1). These strains grew at approximately the same rate in Middlebrook 7H9 broth cultures and displayed different levels of attenuation in bovine macrophages (Lamont et al., 2014; Rathnaiah et al., under review). Some of these mutants have been described elsewhere (Shin et al., 2006; Hines et al., 2014; Settles et al., 2014). The parental strains for these mutants are all K-10 except strain 329, which is the ATCC 19698 strain. Live-attenuated MAP vaccine strains were blinded at Penn State University and received by the laboratory conducting the mouse trial (Cornell University and Oregon State University). The commercial vaccine, Silirum, was purchased and used according to manufacturer recommendations (Pfizer; New York, NY).

**Table 1:** **Mouse treatment groups**.

**Group no**.	**Vaccine**	**Tn insertion**	**Background**	**Treatment**
1	PBS (neg. control)	None	None	PBS-vaccinated, challenged
2	MAP K-10 (Pos. control)	None	K-10	K-10-vaccinated, challenged
3	Silirum (killed vaccine)	None	316F	Vaccinated, challenged
4	Strain 315	MAP1566 (3′ end)	K-10	Vaccinated, challenged
5	Strain 316	Intergenic between MAP3695 and MAP3694c (FadE5)	K-10	Vaccinated, challenged
6	Strain 317	MAP0460	K-10	Vaccinated, challenged
7	Strain 318	Intergenic between MAP0282c andMAP0283c	K-10	Vaccinated, challenged
8	Strain 319	MAP1566	K-10	Vaccinated, challenged
9	Strain 320	Intergenic between MAP2296c andMAP2297c	K-10	Vaccinated, challenged
10	Strain 321	Intergenic between MAP1150c andMAP1151c	K-10	Vaccinated, challenged
11	Strain 329	MAP2408c (FabG2_2)	ATCC 19698	Vaccinated, challenged

### Mice

Four week-old female C57BL/6 mice were obtained from Jackson Laboratory (Bar Harbor, ME) and were acclimated for 2 weeks prior to experimentation. All experiments were performed according to the guidelines of the institutional animal care and use committee at Oregon State University (ACUP #4122) and Cornell University (IACUC #2003-0007).

### Vaccination and challenge of mice

**Figure 1:**
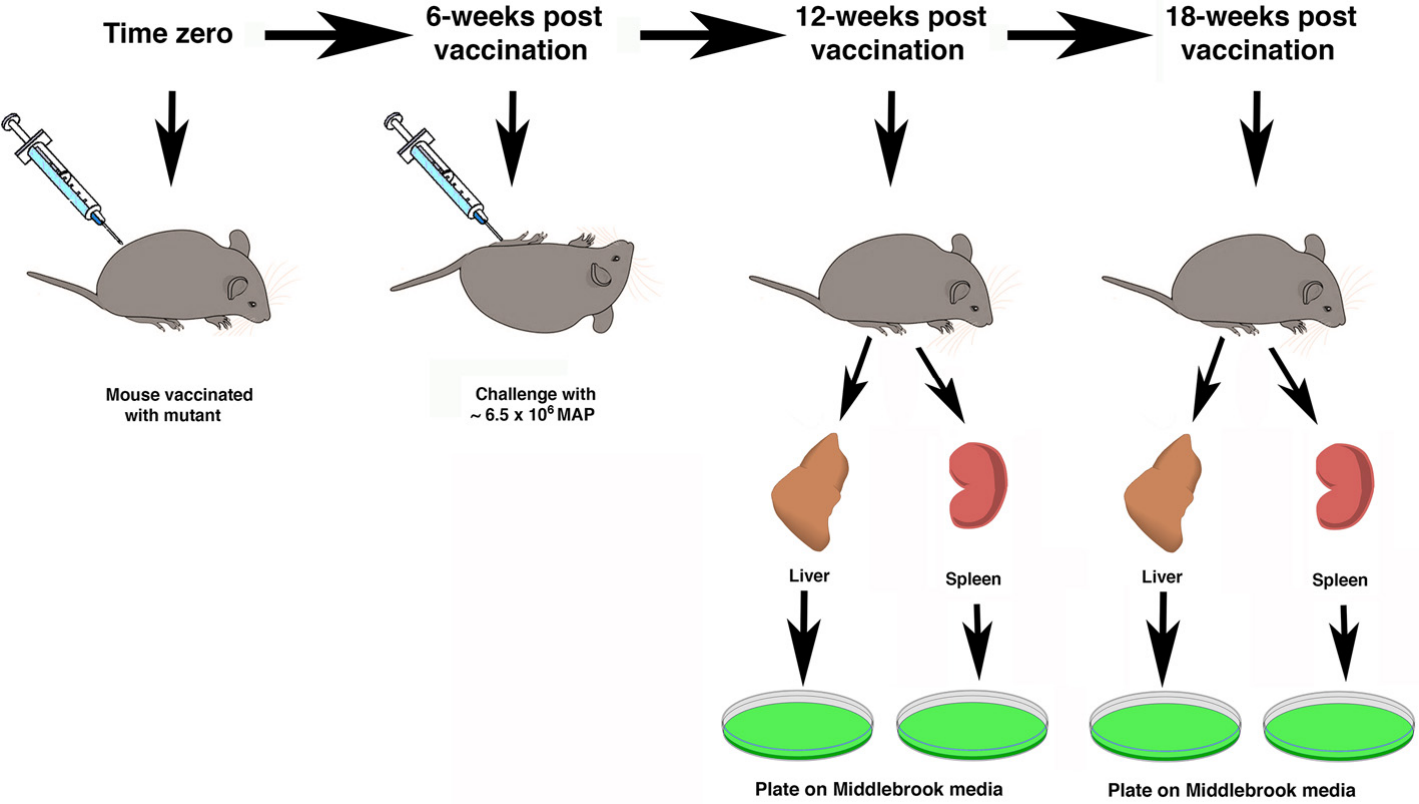
**Schematic overview of study design**. The time scale at the top represents the weeks after vaccination. At time zero mice were immunized with attenuated mutant strains and controls. Mice were challenged by intraperitoneal injection with live MAP 6 weeks after vaccination. Five mice in each of 11 groups were sacrificed at the 12- and 18-week time points. Organs were collected for mycobacterial culture on Middlebrook media (green plates).

The treatment groups for all mice are shown in Table 1. An inoculum for each of the 8 live-attenuated MAP vaccine strains and the wild-type MAP K-10 strain were quantified to an OD_600_ of 0.5 and diluted in the range of 1 × 10^5^–2 × 10^6^ MAP/ml in PBS. Silirum was used in the concentrated form as well as diluted in PBS, and PBS alone was used as a negative control. The manufacturer suggests that the standard bovine dose of Silirum is 1 ml/animal. A calculation based on the average weight of a Silirum vaccinated cow compared to a C57BL/6 mouse suggests the appropriate amount of diluted Silirum vaccine is a 100 μl dose from a 1:1000 dilution of the original Silirum vaccine (final dilution 1:10,000). Thirty mice per group were vaccinated via intraperitoneal injection with 100 μl of live-attenuated or wild-type MAP inoculum prepared as described above (~10^5^ MAP/animal; undiluted and 1:10,000 dilution of Silirum/animal). For quantification, vaccine strains were serially diluted and plated onto Middlebrook 7H11 agar plates alone or supplemented with kanamycin sulfate (100 μg/ml; kan), or hygromycin B (50 μg/ml; hyg) depending on strain requirements. Mice were vaccinated at time zero and then 6 weeks post-vaccination, mice were challenged with 100 μl of MAP K-10 containing ~6.5 × 10^6^ MAP by intraperitoneal injection. Two additional time points were taken post-challenge, each at 6-week intervals. They are the 6-week post challenge (12-week post vaccination) and the 12-week post challenge (18-week post vaccination) time points. The overall study design is summarized in Figure 1.

### Mouse organ and serum collection

At time 0, 6, 12, and 18-weeks post-vaccination blood was collected (5 mice/group/time point) by cardiac puncture. Blood was centrifuged for 15 min at 2500 × *g* and serum was collected and stored at −20°C until ready for antibody analysis. The protective efficacy of each attenuated MAP strain was evaluated by comparing splenomegaly as well as bacterial burden in mouse organs. For CFU enumeration, spleens and livers were collected from mice (5 mice/group/timepoint), weighed, and homogenized using 3-mm stainless steel beads in PBS for 5 min in a Bullet Blender (Next Advance; Averill Park, NY) as per manufacturer's instructions. Homogenates were serially diluted and plated onto 7H11 plates supplemented with polymyxin B (5 μg/ml), carbenicillin (22 μg/ml), and trimethoprim (2 μg/ml) and incubated for 25 days at 37°C. Total CFU on media without kan or hyg and CFU on media with kan or hyg was obtained for each timepoint to distinguish persistence of the vaccine strains (kan^r^ or hyg^r^) vs. protection from the challenge strain (kan^s^ or hyg^s^). The total CFU/organ on media with kan or hyg was subtracted from total CFU/organ on media without the selective antibiotic to obtain the total challenge strain CFU/organ. For groups vaccinated and/or challenged with wild-type MAP K-10 only (Table 1 groups 1, 2, 3), total homogenates were cultured on 7H11 plates without kanamycin or hygromycin and total CFU/organ were used for analysis.

### Statistical analysis

Microsoft Excel software was used to perform Student's *t*-test analyses and analysis of variance. In addition, the Statistical Analysis System (SAS version 9.2; SAS Institute Inc., Cary, NC, 2009) procedure GLM (general linear models) was used to determine if there were differences in CFU counts between groups within each time point (12 and 18 weeks post-vaccination) and organ (liver and spleen). Both the original data (CFU counts) and log-transformed data [log(CFU) counts] were analyzed. In all analyses, differences between groups were considered significant when a probability value of less than 0.05 was obtained.

## Results

### Persistence of map mutants after vaccination of mice

Eight attenuated mutants were included in this study because initial data demonstrated their attenuation in cultured macrophages, which qualified them as candidate vaccines (Wu et al., 2007; Lamont et al., 2014). To further test these live-attenuated strains of MAP, we measured the ability of each strain to initiate systemic infections in mice. Inoculation with Silirum vaccine, K-10 wild-type strain, PBS control, and candidate live-attenuated MAP vaccine strains 315 through 319 resulted in little or no detectable infection in livers and spleens of vaccinated animals at 6 weeks post-vaccination (Table 2). In contrast, candidate vaccine strains 320, 321, and 329 showed notably higher colony counts of MAP in liver and spleen homogenates 6 weeks after vaccination (Table 2). Persistence of these three strains continued out to 12-weeks post vaccination, but no antibiotic resistant colonies were detected by the 18-week time point in the spleen. These data suggest strains 320, 321, and 329 are persistent to at least 12 weeks in the spleen and at least 18 weeks in the liver using this model; however, the other vaccine candidates were clearly attenuated since they could not be cultured even at the earliest time point.

**Table 2:** **Persistence of vaccine strains^a^**.

**Vaccine strain**	**Initial culture conc**.	**Inoculum/mouse**	**Persistence as measured by kan- or hyg-resistant CFUs**		
			**CFUs at 6-weeks^b^**	**CFUs at 12-weeks^b^**	**CFUs at 18-weeks^b^**
PBS (liver)	0	0	0	2760	1120
PBS (spleen)	0	0	0	3380	2570
MAP K-10 (liver)	1.47 × 10^6^	1.47 × 10^5^	0	ND^c^	ND
MAP K-10 (spleen)			3	ND	ND
Strain 315 (liver)	2.8 × 10^4^	2.8 × 10^3^	0	0	0
Strain 315 (spleen)			0	32	0
Strain 316 (liver)	1.8 × 10^5^	1.8 × 10^4^	0	0	0
Strain 316 (spleen)			0	8	0
Strain 317 (liver)	1.26 × 10^6^	1.26 × 10^5^	0	0	0
Strain 317 (spleen)			0	104	0
Strain 318 (liver)	4.2 × 10^5^	4.2 × 10^4^	0	0	0
Strain 318 (spleen)			0	0	0
Strain 319 (liver)	2.1 × 10^5^	2.1 × 10^4^	0	20	0
Strain 319 (spleen)			0	0	0
Strain 320 (liver)	4.2 × 10^6^	4.2 × 10^5^	554	420	34
Strain 320 (spleen)			2180	1410	0
Strain 321 (liver)	2.95 × 10^6^	2.95 × 10^5^	738	20	12
Strain 321 (spleen)			1550	2860	0
Strain 329 (liver)	2.14 × 10^6^	2.14 × 10^5^	476	580	2
Strain 329 (spleen)			206	712	0

a *All values are reported as average CFU/ml among five mice*.

b *All values represent kan- or hyg-resistant colonies except for the PBS control where the K-10 challenge was plated on non-selective media*.

c *ND, not determined. Because K-10 challenge dose could not be distinguished from K-10 vaccination dose, these values could not be obtained*.

### Map colonization of spleen and liver in mice

Six weeks after vaccination the mice were challenged by intraperitoneal injection with wild type MAP (Figure 1). The livers and spleens were collected from mice in each treatment group at the 12- and 18-week time points and cultured for MAP as described in the Materials and Methods section. As a general observation across all treatment groups, spleens had higher MAP burdens as compared to the liver and this difference was significant at the 18-week time point (*P* < 0.05).

#### Liver at 12 weeks post-vaccination

**Figure 2:**
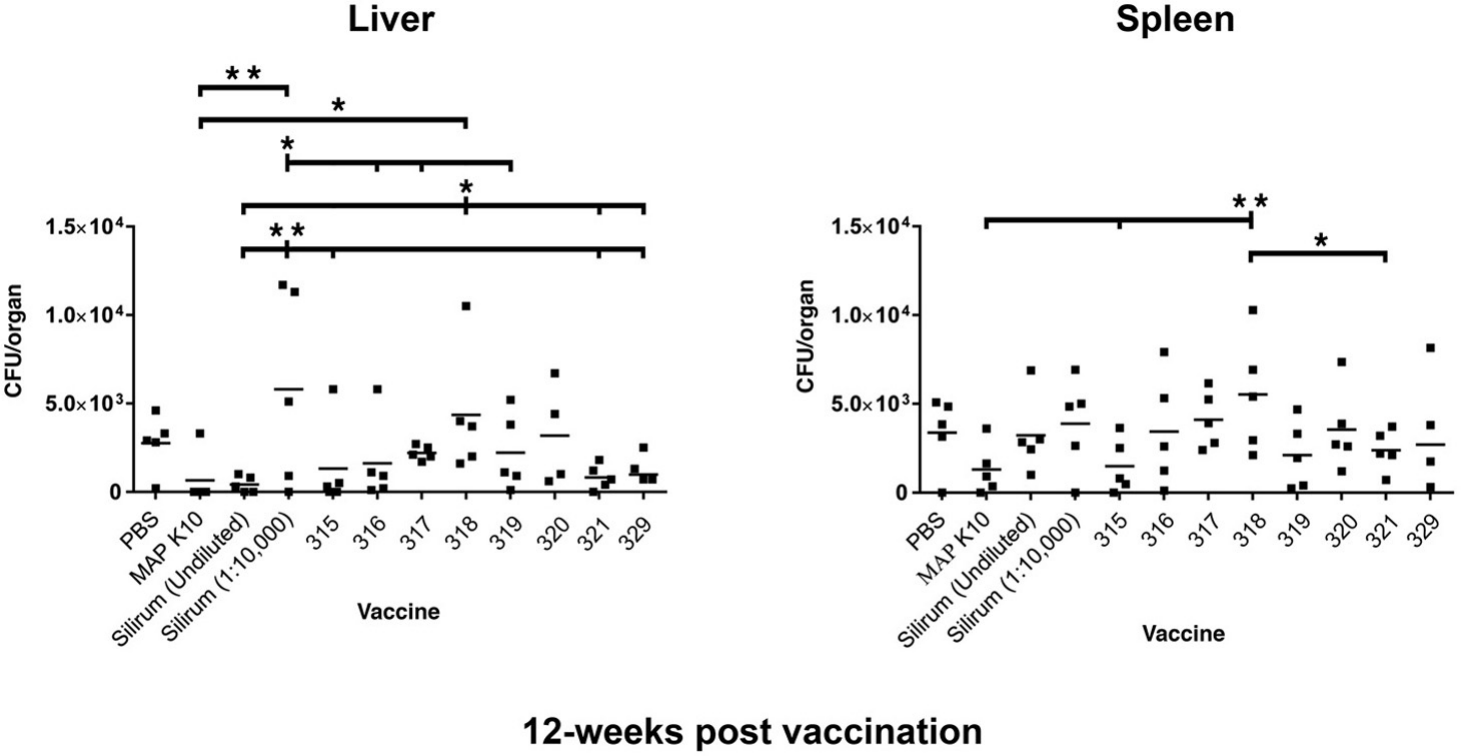
**MAP burden in liver and spleen at 12 weeks post vaccination**. A single asterisk denotes statistical significance at *P* < 0.05 and two asterisks show statistical significance at *P* < 0.01. The bar in each treatment represents the average of five mice.

Culture results for the 12-week time point in liver are shown in the left panel of Figure 2. The K-10 vaccinated group (positive control) had significantly lower CFU counts in the liver than did mice in the diluted Silirum group and strain 318 group. Mice in the diluted Silirum group had significantly higher CFU counts in the liver than did mice in the undiluted Silirum group and all attenuated MAP strains except 318 and 320. These results suggest the diluted Silirum vaccine was too dilute to protect mice from MAP challenge. Mice in the strain 318 group had significantly higher CFU counts in the liver than did mice vaccinated with undiluted Silirum, strain 321 and strain 329. Thus at this time point, strain 315 was statistically the best attenuated vaccine in the liver.

#### Spleen at 12 weeks post-vaccination

Mice in the strain 318 group had significantly higher CFU counts in the spleen than did mice in the K-10 vaccinated group, strain 315 group, and strain 321 group (Figure 2, right panel). In fact, vaccination with strain 318 resulted in the highest MAP burdens among mutant strains in both tissues at the 12-week time point. Strain 315 had the lowest average CFU count among the attenuated MAP mutants.

#### Liver at 18 weeks post-vaccination

**Figure 3:**
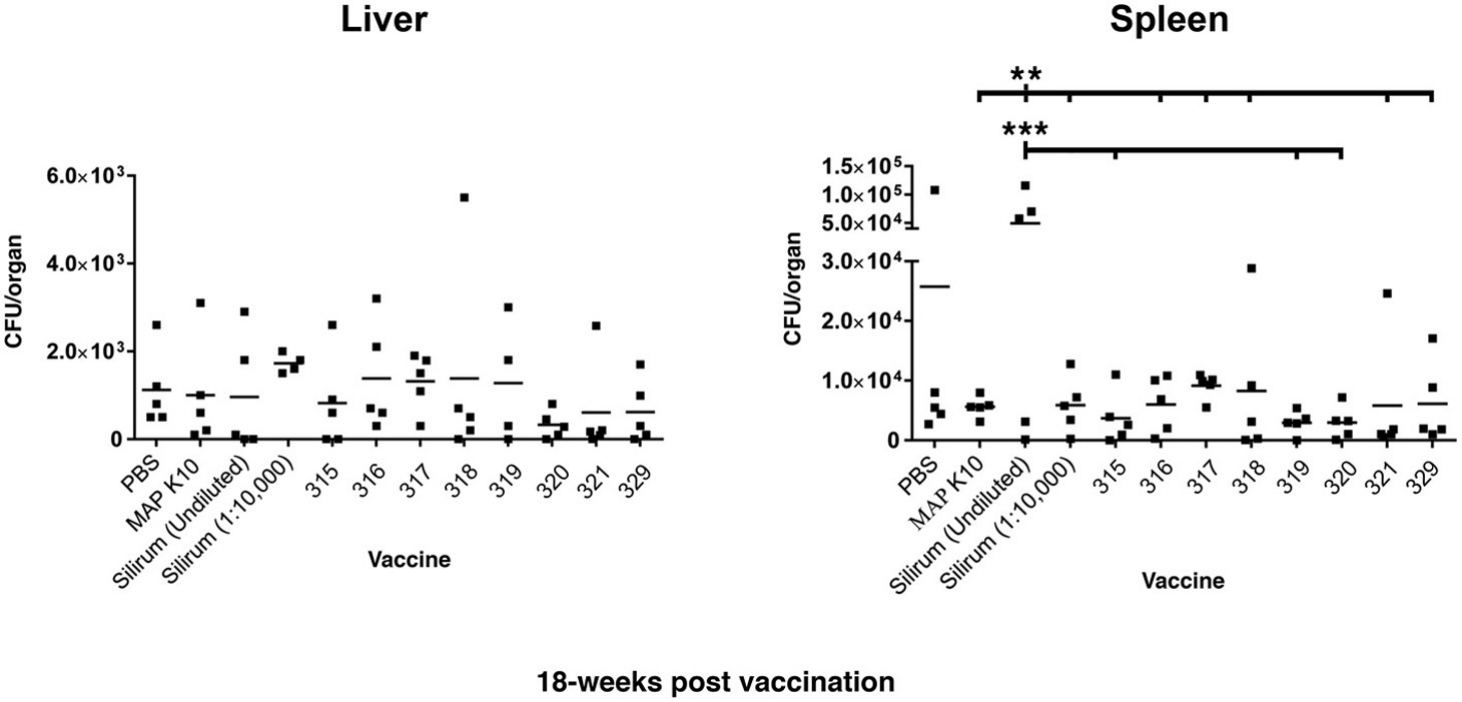
**MAP burden in liver and spleen at 18 weeks post vaccination**. Two asterisks show statistical significance at *P* < 0.01 and three asterisks denote statistical significance at *P* < 0.001. The bar in each treatment represents the average of five mice.

There were no differences in CFU count in the liver between any experimental groups (Figure 3).

#### Spleen at 18 weeks post-vaccination

Mice in the undiluted Silirum group had higher CFU counts in the spleen than did mice in the K-10- vaccinated group, diluted Silirum group, and all the attenuated MAP vaccine treated groups (Figure 3). The undiluted Silirum treatment showed the highest levels of infection seen in the entire study.

Overall, vaccination with strain 319 resulted in low average CFUs in the spleen at both the 12- and 18-week time points but median CFU values in the liver. In contrast to vaccination with strain 315, which showed low CFUs in both tissues at both time points, strain 318 showed the least protection as measured by high bacterial loads in both organs. Both strains 315 and 321 showed equivalent protection against MAP at the 12-week post vaccination time point. However, strain 320 emerged as the best vaccine at the 18-week time point.

### Spleen and liver pathology

**Figure 4:**
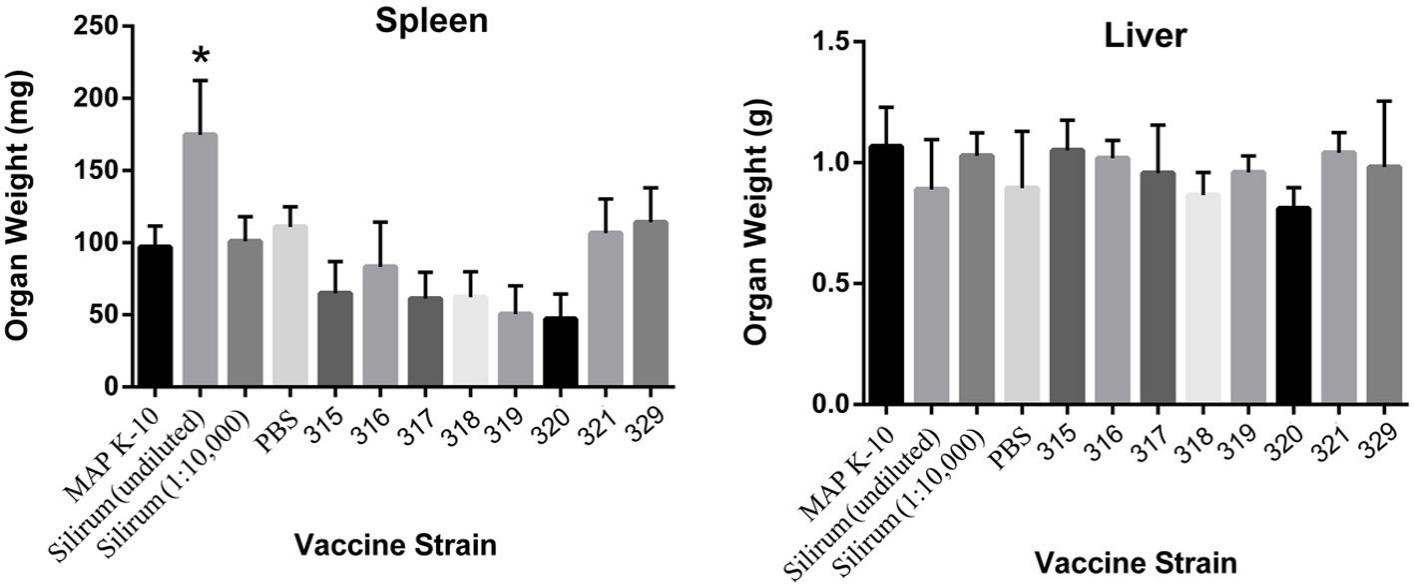
**Induction of splenomegaly in undiluted Silirum vaccinated mice at 18 weeks post vaccination**. The graphs show the average spleen and liver weights of 5 mice per group. Statistically significant differences in spleen size of undiluted Silirum treated mice compared to other treatment groups are indicated by an asterisk (*P* < 0.05 for 329; *P* < 0.01 for 321 and PBS; *P* < 0.005 for K-10, diluted Silirum and 316; *P* < 0.0005 for 315, 317, 318, 319, and 320).

The spleen and liver of each mouse were weighed prior to processing for culture. No significant differences were observed in the liver; however, vaccination with the undiluted Silirum had a significant effect on spleen size (Figure 4). At 18 weeks post vaccination, mice in all other experimental groups had significantly smaller spleens than the undiluted Silirum vaccinated group. The enlarged spleens of this one group demonstrate the strong toxicity or hyper-stimulation obtained with this high-dose whole-cell vaccine.

## Discussion

The development of new live MAP vaccine candidates is still in its early stages, yet through a three-phase vaccine trial using available attenuated mutants, several key insights can be obtained. The results will enable a unique look at how mouse vaccination and challenge data will predict protection in a ruminant model, since a selection of these vaccine strains were moved forward into a goat vaccine trial (Hines et al., 2014). In addition, a determination can be made of whether killed whole-cell vaccines, which are the current formulation in commercially available vaccines against Johne's disease, vs. live attenuated strains are better vaccines for Johne's disease. Here we showed that different live attenuated strains protect with varying degrees (ranked in order of lowest bacterial burden in both tissue combined to highest 315, 319, 321, 320, 329, 316, 317, and 318) against tissue infection in a mouse model, while the whole-cell vaccine Silirum, in the conditions tested, yielded poor protection either as diluted or undiluted formulations. This is similar to what was observed when comparing the live *leuD* mutant to the killed Mycopar vaccine. That study showed the *leuD* mutant was more protective against MAP challenge in goats (Faisal et al., 2013). The *leuD* mutant also induced a protection against MAP challenge in a mouse model (Chen et al., 2012). However, there are some differences between these two studies that make comparisons difficult. The latter study used a different bacterin (Mycopar derived from strain 18) and inoculation route (subcutaneous).

Nonetheless, these insights will be helpful when new MAP vaccine strains become available. For many years, it has been suggested that an effective anti-mycobacterial vaccine strain must replicate in the host tissue in order to induce protective immune responses. While this is the case for BCG vaccination against *Mycobacterium tuberculosis* infection, it remains an issue needing further experimentation for MAP vaccination. The trial in mice reported herein seems to indicate otherwise, but results in goats (Hines et al., 2014) are consistent with this hypothesis. Moreover, it is noted that while experimental vaccines were inoculated intraperitoneally in the current mouse trial, goats were vaccinated with a subset of these attenuated mutants and challenged by the oral route (Hines et al., 2014). Thus, factors other than animal species may contribute to the observed differences.

Our study measured both persistence of the vaccine strains as well as infection levels of the challenge strain in the spleen and liver. These two organs are primarily used to assess systemic implantation and organ compromise for MAP in the mouse at 6 and 12 weeks post challenge (Shin et al., 2006; Wu et al., 2007) although lymph nodes and intestinal tissues have also been cultured (Huntley et al., 2005; Shin et al., 2006; Cooney et al., 2014). Although not part of our study design, a recent paper suggests lymph nodes are a good tissue to track progression of MAP infection in the murine model (Cooney et al., 2014). Nonetheless, we observed slightly higher infection levels in the spleen as compared to the liver, which is commonly seen and may represent the result of greater vascularization in the spleen. We also observed three mutant strains that were able to persist for up to 12 weeks post vaccination, but were cleared by 18 weeks post vaccination. This is similar to that observed with the *fabG2_2* and *impA* mutants in a separate study (Shin et al., 2006). In fact, the *fabG2_2* mutant from that study is the same as strain 329 in the current study, thus confirming persistence for this mutant. Because these three mutants did not clearly outperform the more attenuated strains, it remains uncertain whether these more persistent mutants result in better protection from implantation and infection than fully attenuated mutants. It has been postulated previously that MAP mutants, which cannot survive in mouse peritoneal macrophages, might be a reason for the inability of attenuated strains to colonize mouse organs (Shin et al., 2006).

In our mouse model, the diluted Silirum vaccine demonstrated poor protection relative to the attenuated vaccine strains. The vaccine was designed for sheep and the manufacturer recommends 1 ml/ovine dose and thus 0.1 ml/mouse might be a reasonable empirical dosage, but there is no experimental data to verify this as an appropriate dose. Therefore, we chose to use Silirum undiluted and at a 1:10,000 dilution in this study. It was observed that any protective effect in the liver could be diluted out, since average numbers of colony forming units were lower in the undiluted Silirum relative to the 1:10,000 dilution. However, this trend did not hold true for either time point in the spleen. There were unusually high numbers of colony forming units in three of the five mice vaccinated with undiluted Silirum (Figure 3, right panel). In contrast, this same vaccine has been shown to be very effective at reducing mortality and delaying fecal shedding in Merino sheep (Reddacliff et al., 2006). The amount of Silirum vaccine given per mouse could have had a detrimental effect in a variety of ways. As the amount given to a mouse was more in proportion to what a farm animal would receive, it is possible that the components (oil, MAP antigens, etc.) of the vaccine inhibited its ability to fight off infection over the length of the trial. The undiluted vaccine could have had a toxic effect on the spleen, which may explain the enlarged spleens in the undiluted Silirum vaccinated group. This effect may have led to a higher level of colonization by the MAP challenge strain later during infection. It also might simply suggest that the mouse may not be a good predictive model for vaccines against Johne's disease.

This is not a comprehensive list of attenuated mutants in MAP. Unfortunately, the *leuD* mutant was not included in these studies. It has recently shown efficacy against MAP challenge in both a mouse and goat model (Chen et al., 2012; Faisal et al., 2013). However, this is the only published attenuated mutant of MAP that was not enrolled in the Johne's Disease Integrated Program's three-phase vaccine trial.

The wild-type challenge strain did not appear fully virulent in the mouse model. The K-10 strain used in this mouse study is the same strain that was used to construct most of the mutants. However, as has been clearly shown for *M. tuberculosis* (Ioerger et al., 2010), identical strains kept in different laboratories diverge over time. Therefore, the apparent slight attenuation of the wild type strain in the mouse may just reflect the natural passage history in two laboratories. Another possibility is that when inoculated at low dose it may just be that some mutants grew better at this time point, but if the time points were extended, their attenuation would be clearer.

The mouse model is used to understand invasion, virulence mechanisms, and evaluate vaccine strains, but it is limited in its ability to develop the full disease observed with MAP infection in ruminants. Due to this pitfall of the model, it is possible that wild-type MAP may provide similar, early protection as attenuated strains against subsequent infection. This is likely due to the common components in the whole cell bacterial vaccines of live-attenuated and non-attenuated strains. However, when translated to a caprine or bovine challenge model, this protection is abrogated as the wild-type virulent MAP strain is capable of progressing to the clinical stages of Johne's disease. The *in vitro* trials were for virulence and survival in macrophages, and so we hypothesized that the live-attenuated strains would be unable to progress to full infection in a ruminant model. The five selected mutants in our study were entered into a goat trial to examine which mutant is the best performer in a ruminant model. Analysis of these data are reported elsewhere (Hines et al., 2014) and results seem to suggest a low level of correlation, indicating that the mouse trial, as conducted, has a relatively poor predictive value for the results in a ruminant host such as goats. Alternatively, oral vaccination may not be the best route to evaluate attenuated strains in goats.

### Conflict of interest statement

The authors declare that the research was conducted in the absence of any commercial or financial relationships that could be construed as a potential conflict of interest.
